# Effects of Apple (*Malus pomila*) Pomace-Derived Pectin on the Innate Immune Responses, Expressions of Key Immune-Related Genes, Growth Performance, and Digestive Enzyme Activity of Rainbow Trout (*Oncorhynchus mykiss*)

**DOI:** 10.3390/ani11072117

**Published:** 2021-07-16

**Authors:** Seyed Hossein Hoseinifar, Ghasem Rashidian, Hamed Ghafarifarsani, Mohammad Amin Jahazi, Mehdi Soltani, Hien Van Doan, Ehab El-Haroun, Marina Paolucci

**Affiliations:** 1 Department of Fisheries, Faculty of Fisheries and Environmental Sciences, Gorgan University of Agricultural Sciences and Natural Resources, 49189-43464 Gorgan, Iran; jahaziamin@gmail.com; 2 Department of Aquaculture, Faculty of Natural Resources and Marine Sciences, Tarbiat Modares University, Noor 4641776489, Iran; ghasemrashidiyan@gmail.com; 3 Department of Fisheries, Faculty of Agriculture and Natural Resources, Urmia University, 5756151818 Urmia, Iran; hamed_ghafari@alumni.ut.ac.ir; 4 Department of Aquatic Animal Health Faculty of Veterinary Medicine, University of Tehran, 1419963111 Tehran, Iran; msoltani@ut.ac.ir; 5 Centre for Sustainable Aquatic Ecosystems, University of Murdoch, Murdoch, WA 6150, Australia; 6 Department of Animal and Aquatic Sciences, Faculty of Agriculture, Chiang Mai University, Chiang Mai 50200, Thailand; hien.d@cmu.ac.th; 7 Fish Nutrition Research Laboratory, Department of Animal Production, Faculty of Agriculture, Cairo University, Cairo 12411, Egypt; elharoun@gmail.com; 8 Department of Sciences and Technologies, University of Sannio, 82100 Benevento, Italy; paolucci@unisannio.it

**Keywords:** apple pomace, pectin, prebiotic, rainbow trout, immune genes, innate immunity, growth performance

## Abstract

**Simple Summary:**

The present study investigated possible administration of pectin derived from apple pomace as a beneficial and cost-effective feed additive to be used in trout culture. To achieve the aim, a range of parameters were measured including immune parameters (both at physiological and molecular levels), growth performance and digestive enzyme activity. The results showed promising effects on the measured parameters and hence we can suggest administration of this feed additive in trout culture.

**Abstract:**

Pectins are a group of carbohydrates found in structural parts of terrestrial plants with wide industrial and biomedical applications. This study was designed to investigate the dietary effects of apple pomace-derived pectin (APDP) in rainbow trout (*Oncorhynchus mykiss*). Four formulated diets were provided with different inclusion levels of APDP for 30 days: 0, 5, 10, and 20 g kg^−1^; referred to as control, P1, P2, and P3, respectively. In this study, 300 fish (3.56 ± 0.007 g) were randomly distributed into twelve fiberglass tanks and fed 3% of their respective body weight four times a day. At the end of the experiment, growth parameters, including weight gain, specific growth rate, and food conversion ratio (FCR) were significantly improved in P1 and P2 treatments compared to those of the other treatments. Results from proximate composition analysis showed that protein content increased, and lipid decreased in the P2 and P3 groups. Serum lysozyme, complement activity, total immunoglobulin levels, and total protein were significantly enhanced in all treatments compared to those of the control group. Gene expression results showed no significant difference in regulation of interleukin-1β (IL-1β); however, up-regulation of lysozyme, interleukin-8 (IL-8), and tumor necrosis factor-α (TNF-α) was observed in both P1 and P2. Unlike lipase, the activity of protease and amylase significantly increased in fish receiving different levels of APDP compared to the control (*p* < 0.05). In conclusion, the present findings suggest APDA as a promising feed additive for rainbow trout.

## 1. Introduction

The consumption of fish provides mankind with one of its healthiest foods; it is rich in calcium, phosphorus, and other minerals. The demand for fish will likely continue to increase as our population grows [1]. In modern aquaculture, fish are fed with artificial diets, thereby creating the need to carefully formulate diets that directly influence the quality of the final product [2]. One of the main obstacles challenging the sustainable development of aquaculture is the loss of yields, due to infectious disease and growth retardation caused by inevitable stress when fish are reared in high stocking densities. It is possible, under the same confinements, to enhance fish growth and immunity using feed additives to ensure efficient and profitable production along with a prosperous and high-quality product. Thus, there has been a growing interest among researchers to find and evaluate potentially effective substances that have a beneficial effect upon fish growth and health, in order to address the above-aforementioned issues [3,4,5,6,7,8,9,10,11,12].

Fibers, which are carbohydrates that cannot be hydrolyzed by fish digestive enzymes or absorbed by the small intestine, are included at roughly 7% in typical fish diets [13]. Pectin is classified as a soluble fiber, within a group of naturally occurring polymers acting as structural materials in all terrestrial plants. Pectin can be fermented by fish microbiota and may give rise to short-chain fatty acids [13]. Prebiotics, on the other hand, are defined as non-digestible carbohydrates, which may enhance fish growth [14] and immunity [15,16,17,18,19,20,21].

Pectin, derived from various sources, has been often tested for its prebiotic potential in different fish species [22,23,24,25]. It is likely that the initial source of pectin, target fish, administration method, and levels of inclusion strongly affect its effectiveness. Pectin is also used as a gelling and stabilizing agent in the food and cosmetic industries, shown to have multiple, positive effects on human health, including lowering cholesterol and serum glucose levels, reducing cancer [26], and stimulating the immune response [27]. There is limited literature available regarding the dietary effects of pectin in aquatic animals. Thus, the present study aimed to evaluate the prebiotic potential of apple pomace-derived pectin on growth performance, digestive enzyme activity, innate immune responses, and the expressions of key immune genes of rainbow trout.

## 2. Materials and Methods

### 2.1. Pectin Extraction Procedure

Apple pomace was procured from a local juice company in Gorgan, Iran. Afterward, 200 g of wastes were treated with 400 mL of distilled water, and the pH was set at 1.5 using citric acid. The resulting mixture was blended for ten minutes, heated in a microwave (700 W), and then stored at 85 °C for five minutes. The mixture was then allowed to cool down at room temperature and thoroughly shook for 15 min to achieve maximum interaction. Resulting mixture was passed through cleaning tissues followed by addition of 96% ethanol. The mixture was passed through a cleaning tissue, followed by the addition of 96% ethanol. The resultant cloud-like gel was separated and condensed at 4 °C for one hour. To remove the solvent, the gel was centrifuged at 10,000 rpm for ten minutes, and the precipitate was twice washed with 96% ethanol and dried at 40 °C. The powdered samples were packed in plastic bags and kept at 4 °C for further use.

### 2.2. Formulation and Preparation of the Experimental Diets

A basal diet was formulated based on the standard nutritional requirements of rainbow trout (Table 1). Cellulose in the basal diet was substituted with zero (control), 5, 10, and 20 g of pectin, referred to as the control, P1, P2, and P3, respectively. The basal ingredients were mixed with water to achieve a smooth dough. The dough was then passed through an industrial grinder, and the diets were dried overnight at 40 °C. The pellets were sifted through a 1 mm mesh and stored in plastic bags at 4 °C until needed.

### 2.3. Experimental Design and Fish Husbandry

The experiment was carried out in a completely randomized design with four treatments in triplicates. Fish were supplied from a local farm and transferred to the aquaculture laboratory at Gorgan University, where they adapted to lab conditions for two weeks. During acclimatization, fish were fed on basal diet (control diet) four times a day and were inspected for physical injuries and general (visible) health issues. Three hundred healthy rainbow trout fingerlings weighing 3.56 ± 0.007 g were stocked in 12 fiberglass tanks and fed the experimental diets at 3% of their respective biomass, four times a day, for 30 days. In the third week, the fish were weighed, mortalities were recorded, and the feeding regime was adjusted accordingly. Rearing water quality indicators, including pH, dissolved oxygen, and temperature, were monitored daily with a portable multi-meter, (HACH, Loveland, Colorado, USA) and maintained at 7.5–8.2, 7–8 mg L^−1^ and 16 ± 2 °C, respectively. Water exchange was set as 180% with fresh aerated water every day. The tanks were siphoned for feed leftovers and fecal matter. Photoperiodwas maintained (12 light and 12 dark) using artificial light.

#### Sampling

At the end of the experiment, nine fish from each treatment were randomly selected and sampled to evaluate growth performance, body composition, digestive enzymes activity, and serum immunological parameters. Rainbow trout were anesthetized with clove powder (100 mg L^−1^). Blood samples were collected from the caudal vein using both heparinized and non-heparinized syringes with 20-gauge needles, kept for four hours at 4 °C, and then centrifuged to obtain serum samples. The collected plasma and serum were stored at −80 °C for further analysis. To study the expression of immune and inflammation-related genes, the head kidney was sampled, snap-frozen in liquid nitrogen, and kept at −80 °C until needed. The digestive tract was removed in whole, and the hepatosomatic index (HSI) and viscerosomatic index (VSI) were recorded. The sampling process included the evaluation of the digestive enzymes. To measure the activity of the digestive enzymes, fish were sampled from each replication. After dissection, samples of the middle intestine were immediately homogenized in ten volumes (*w*/*v*—1198) of ice-cold physiological saline solution and centrifuged at 5000× *g* for 15 min at 4 °C. The supernatant was then stored for endogenous enzyme activity analysis, following the protocol designed by [22].

### 2.4. Growth Parameters

At the end of the experiment, fish were starved for 24 h and sedated with 100 mg L^−1^ clove powder. The growth parameters, including final weight (FW), weight gain (WG), survival rate (SR), specific growth rate (SGR), food conversion ratio (FCR), hepatosomatic index (HSI), and viscerosomatic index (VSI) were measured according to the following equations:

WG (g) = (Wf − Wi)/Wi(1)

SGR (% day-1) =100 × (ln Wf − ln Wi)/t(2)

FCR = FI (g)/wet WG (g)(3)

SR (%) = (Nf × 100)/Ni(4)

HSI (%) = 100 × [liver weight (g)/wet weight (g)](5)

VSI (%) = 100 × [viscera weight (g)/whole fish weight (g)](6)

In these equations, Wf and Wi are final and initial weight, respectively, t is the experiment duration, FI stands for food intake, Nf and Ni represent the final and the initial number of fish.

### 2.5. Digestive Enzymes Activity

Gut samples were homogenized in HCl-tris (50 mM) buffer in a 1 to 5 ratio (*v*/*v*) for 90 s. The homogenate was centrifuged at 9000× *g* for 20 min at 4 °C and then stored at −80 °C until the activities of the digestive enzymes were measured according to [28].

#### 2.5.1. Amylase Activity

Starch decomposition under the influence of the enzyme to produce maltose was measured by colorimetric method and color intensity change in the dinitrosalicylic acid reagent. In brief, and aliquot of 250 μL of enzyme extract was added to the test tubes and incubated for three to four minutes at 25 °C to reach equilibrium temperature. Then, 250 μL of 1% starch was added to the tubes and incubation continued for three minutes. Afterward, 0.5 mL of dinitrosalicylic acid dye was added to the tubes, and the tubes were placed in a boiling water bath for five minutes, and then cooled to room temperature. In the next step, 5 mL of distilled water were added to the tubes and subsequent absorption was read at 540 nm. The standard maltose curve was used to measure the amount of maltose released by the enzyme. The unit of amylase activity was calculated in terms of maltose released by enzyme per unit time (min) per milligram.

#### 2.5.2. Lipase Activity

The lipase activity in the crude enzyme extract was determined by hydrolysis of p-nitrophenylemyristate through the spectrophotometry method. For each assay of lipase activity, 6 μL of the enzyme extract was added to 86 μL of sodium cholate buffer solution and 2.5 μL of methoxyethanol solution. Then 5.5 μL of paranitrophenol meristate substrate were added to the mixture, the resultant solution was incubated at 30 °C for 15 min, and the adsorption was read at 405 nm by spectrophotometer.

#### 2.5.3. Total Protease Activity

A solution of azocasein 1.5 % in 50 mM Tris-Hcl buffer with pH = 7.5 was used as substrate. Then, 20 μL of crude enzyme extract with 0.5 mL of 1.5 % azocasein were incubated in the Tris-Hcl buffer for ten minutes at 25 °C. After incubation, 0.5 mL of TCA (trichloroacetic acid) solution was added to terminate the reaction. The microtubes were then centrifuged at 6500× *g* for five minutes. Lastly, the top solution of each microtube was transferred into a purification microplate, and their absorbance was read by spectrophotometry at 440 nm. Protease-specific activity was calculated according to the incubation period (10 min) and the amount of enzyme extract protein (mg).

#### 2.5.4. Measurement of Soluble Protein Concentration

Soluble protein of crude pyloric extract of rainbow trout was measured according to the method described by [29], in which Bovine serum albumin (BSA) was used as a standard. To measure the protein of the crude enzyme extract solution, 5 μL of diluted enzyme extract and 250 μL of the Bradford reagent were added. After seven minutes of incubation at 25 °C, the optical absorption at 595 nm was recorded.

### 2.6. Serum Immunological Parameters

#### 2.6.1. Total Serum Immunoglobulin (Total Ig)

The serum total immunoglobulin levels were measured according to [30], by determining the total serum protein before and after immunoglobulin precipitation, using a 12% polyethylene glycol solution.

#### 2.6.2. Serum Lysozyme Activity

Serum lysozyme activity was measured according to [31], which is based on the lysis of *Micrococcus luteus*, a Gram-positive bacteria sensitive lysozyme.

#### 2.6.3. Serum Complement Activity (ACH50)

ACH50 activity is another nonspecific immune parameter studied in this experiment, which was measured using hemolysis of sheep red blood cells through the method described by [32]. Erythrocytes were washed three times with ethylene glycol tetraacetic acid-magnesium-gelatin vernal buffer and the number of cells was adjusted by neobar slide per mL of 2 10 108 buffer cells. The serum samples were diluted 100 times with the buffer. Red blood cells (100 μL) were then added to each tube and after incubation at 20 °C for 90 min, and 3.15 mL of sodium chloride solution were added to each tube. The product was centrifuged at 2500 rpm and, lastly, via spectrophotometer, the absorption of the supernatant was read at a wavelength of 414 nm. The volume of serum and mucus that caused 50% hemolysis was considered the complement activity of the sample, which was calculated using the following equation:

ACH50 (U/mL) = K × 0.01 × 0.5(7)

In which K is the volume of serum/mucus in mL causing 50% hemolysis. 0.5 is a fixed number, and 0.01 is the dilution factor in this test, as the solution is diluted 100 times.

#### 2.6.4. Total Serum Protein

Determination of total serum protein was measured by colorimetric method using a commercial kit (Pars Azmun, Iran) following the manufacturer’s instructions.

### 2.7. Gene Expression

Total RNA was extracted using RNX-Plus (Sinaclon, Tehran, Iran) according to the manufacturer’s instructions. The quantity and quality of RNA were checked using a spectrophotometer and 1.5% agarose gel electrophoresis, respectively [33,34]. Total RNA (1.00 μg) was used to construct cDNA (Sinaclon, Tehran, Iran), which was stored at −20 °C for further analyses. The cDNA was subjected to real-time PCR using 2X SYBR Green PCR Master Mix (Sinaclon, Tehran, Iran).

The real-time PCR condition of all genes followed the standard protocol developed by [33]. Primers (Table 2) were designed according to the cDNA sequences of rainbow trout in GenBank^®^ [35,36,37]. Three technical replicates for each sample were performed and the threshold cycle (CT) was determined for each run. Gene expression levels were normalized to β-actin. The average ± SEM values of the cycle threshold (Ct) of beta-actine of was 25 ± 1.35. The relative mRNA expression of the genes was quantified by the 2 –ΔΔCt method after modifications, in that the primers were amplified with an efficiency of from 97% to 99% [38]. Data were analyzed using the iQ5 optical system software version 2.0 (Bio-Rad).

### 2.8. Statistical Analysis

The present experiment was performed in a completely randomized design. First, the normality of the obtained data was tested using Kolmogorov–Smirnov test. Statistical analysis of data was performed through SPSS 20 software using one-way ANOVA and Tukey’s HSD test. All assumptions of analysis of variance were studied before statistical analysis, and all tests were interpreted at a significance level of *p* < 0.05. Results are reported as mean ± SE. Excel software 2016 was also used to draw the diagrams.

## 3. Results

### 3.1. Growth Performance

The results related to growth parameters, are shown in Table 3, indicate that fish fed diets supplemented with pectin significantly increase the FW and SGR compared to that of the control diet (*p* ≤ 0.05). However, fish fed either P1 or P2 showed significantly increase FW, and WG compared to fish fed 20 g kg^−1^ APDP. The results further showed that the 10 g inclusion of APDP improved FCR compared to all other groups (*p* < 0.05). Fish fed the pectin supplemented diets recorded 100% survival rate versus 97.77%. of the control Additionally, the highest and the lowest of HSI and VSI values were found in both the control and P2 treatments (*p* < 0.05).

### 3.2. Body Composition

The results for proximate body composition, shown in Table 4, showed no significant differences among treatments in terms of moisture, crude protein, crude lipid, or ash contents.

### 3.3. Digestive Enzymes

The results of the analysis of digestive enzyme activity are shown in Table 5. Fish fed diets supplemented with pectin significantly increased protease and amylase activity (*p* < 0.05) compared to that of the control diet. No significant differences were noticed among treatments in terms of lipase activity; however, the highest amount of lipase activity was observed in fish fed both 0 g (control) and 20 g of pectin in the diet.

### 3.4. Immunological Parameters

**Figure 1:**
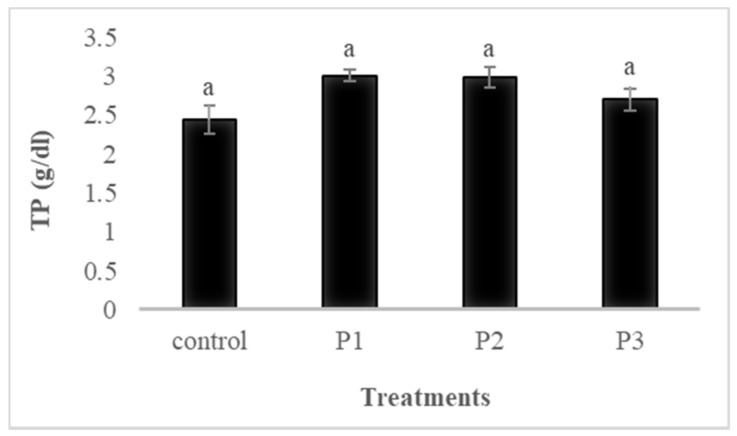
The effects of dietary pectin on serum total protein of rainbow trout fed four experimental diets with different inclusion levels control: 0, P1: 5, P2: 10, and P3: 20 g of apple pomace derived pectin (APDP) for 30 days. Bars assigned with same superscripts are not significantly different (*p* > 0.05); values are presented as the mean ± SE. (*n* = 9).

**Figure 2:**
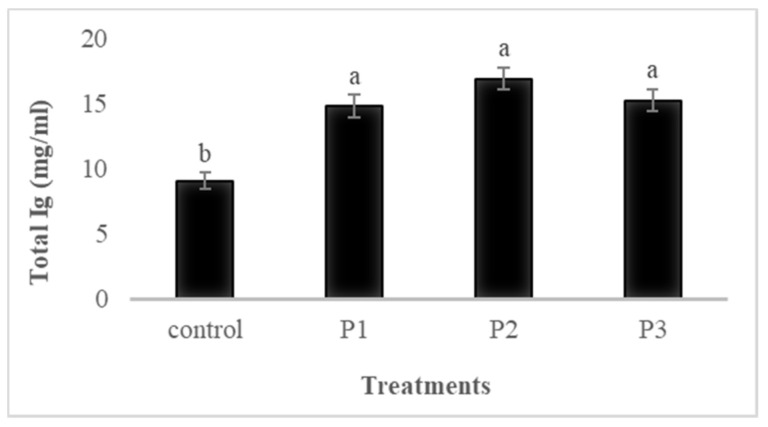
The effects of dietary pectin on serum total immunoglobulin level of rainbow trout fed four experimental diets with different inclusion levels control: 0, P1: 5, P2: 10, and P3: 20 g of apple pomace derived pectin (APDP) for 30 days. Bars assigned with different superscripts are significantly different (*p* < 0.05); values are presented as the mean ± SE. (*n* = 9).

**Figure 3:**
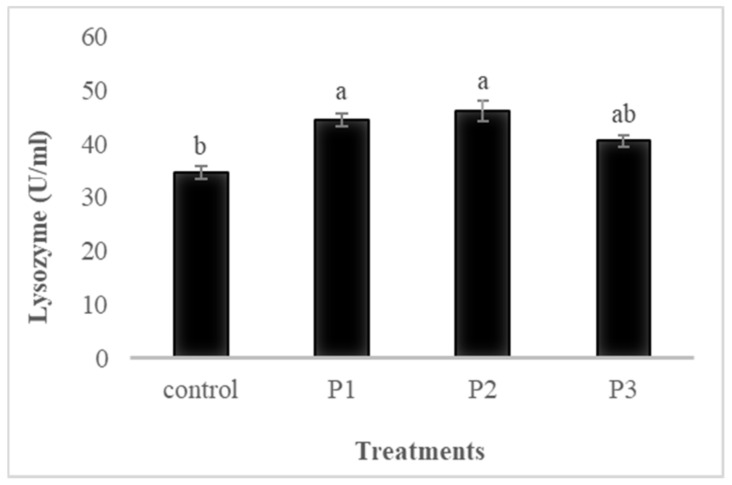
The effects of dietary pectin on serum lysozyme activity of rainbow trout fed four experimental diets with different inclusion levels control: 0, P1: 5, P2: 10, and P3: 20 g of apple pomace derived pectin (APDP) for 30 days. Bars assigned with different superscripts are significantly different (*p* < 0.05); values are presented as the mean ± SE. (*n* = 9).

**Figure 4:**
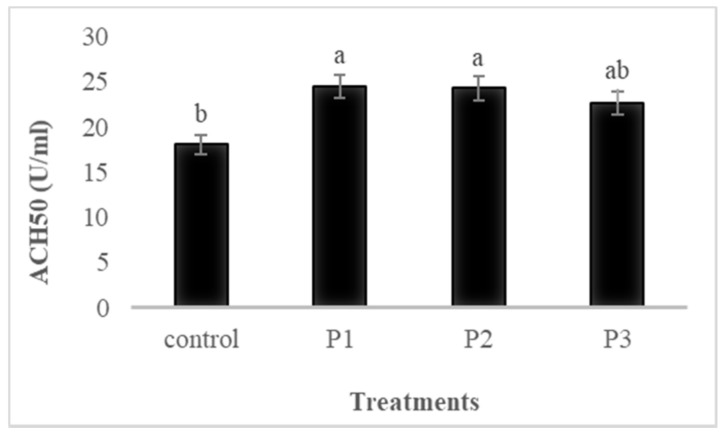
The effects of dietary pectin on serum alternative hemolytic complement activity (ACH50) of rainbow trout fed four experimental diets with different inclusion levels control: 0, P1: 5, P2: 10, and P3: 20 g of apple pomace derived pectin (APDP) for 30 days. Bars assigned with different superscripts are significantly different (*p* < 0.05); values are presented as the mean ± SE. (*n* = 9).

Serum immunological parameters of fish fed the four experimental diets for 30 days are shown in Figure 1, Figure 2, Figure 3 and Figure 4.

#### 3.4.1. Total Protein (TP)

The results showed that the serum total protein tended to increase with the inclusion of pectin, although no significant difference was observed among treatments (*p* ≥ 0.05; Figure 1). However, the pectin treatments (P1, P2, and P3) did not influence serum total protein contents.

#### 3.4.2. Total Ig

The results of total immunoglobulin (total Ig) in each group are shown in Figure 2, which indicates that total Ig significantly increased in the groups fed supplementary pectin compared to that of the control (*p* < 0.05), yet there were no significant differences (*p* > 0.05) among the pectin groups (*p* > 0.05).

#### 3.4.3. Lysozyme

Fish fed diets supplemented with 10 and 20 g of APDP showed higher values (*p* ≤ 0.05) of serum lysozyme compared to the other diets (Figure 3). No differences were detected between the P2 and P3 groups; however, among the pectin treatments, the inclusion of 10 g kg^−1^ of pectin recorded the highest lysozyme value.

#### 3.4.4. ACH50

Fish fed diets supplemented with 5, 10, and 20 g of APDP showed higher values (*p* ≤ 0.05) of ACH50 compared to that of the control group (Figure 4). No differences were detected between P1, P2, and P3 (*p* > 0.05).

### 3.5. Gene Expression

**Figure 5:**
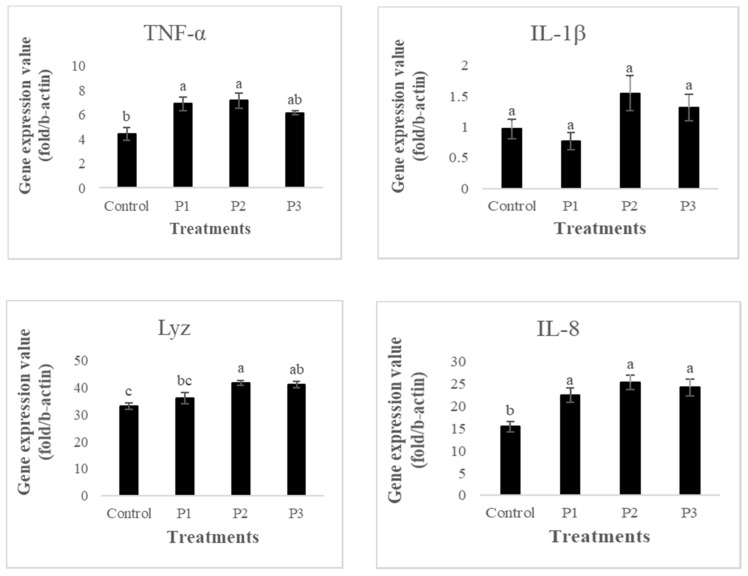
The relative expression of immune related genes, TNF-α, IL-1β, Lyz, and IL-8, in head kidney of rainbow trout fed four experimental diets with different inclusion levels control: 0, P1: 5, P2: 10, and P3: 20 g of apple pomace derived pectin (APDP) for 30 days. Values are presented as the mean ± SE. (*n* = 9). Different letters (a–c) in each row indicate significant differences (*p* < 0.05).

The inclusion of pectin in the fish diets improved the TNF-α gene expressions compared to that of the control group (*p* ≤ 0.05; Figure 5). The mRNA levels in the groups fed with 5 and 10 g of pectin supplementation were higher than that of the 20 g group (*p* > 0.05), with no significant differences noted between P1, P2, and P3.

The present study showed that no statistically significant differences (*p* ≥ 0.05; Figure 5) in IL-1β gene expression between any treatment and the control.

The present study showed that fish fed diets supplemented with pectin increased the lysozyme gene expression in the anterior kidney tissues compared to the control group (*p* < 0.05; Figure 5); however, there were no significant differences among control and 5 g pectin diet (*p* > 0.05).

In the case of IL-8, the addition of pectin to the experimental diets affected the expression of the IL-8 gene, in which the levels of IL-8 mRNA in the 5, 10 and 20 g pectin inclusion groups significantly increased compared to the control treatment (*p* < 0.05; Figure 5).

## 4. Discussion

Modern aquaculture contributes significantly to human food security with a host of enjoyable, high-quality products. It is, therefore, crucial to direct research activities towards refining aquafeeds and improving fish growth and health using safe, functional additives. Researchers [7,19,21] have reached a consensus regarding the beneficial effects of prebiotics to enhance fish growth and immunity, due, perhaps, to the modulation in gut microbiota and increasing nutrient availability.

The present findings showed that fish fed diets supplemented with pectin significantly increased FW, and SGR, as well as improved FCR compared to the control diet. However, fish fed the 5 g kg^−1^ or 10 g kg^−1^ diets significantly improved FW, WG, and SGR, compared to fish fed 20 g kg^−1^ APDP. Similar results were seen by other researchers using other sources of pectin [22,24]. Goulart et al. [23] tested pectin (derived from citrus pulp) on jundia (*Rhamdia quelen*), and reported orthogonal comparisons where WG, FCR, and HSI were significantly different from those found in the control. In the present study, HSI decreased remarkably in all fish receiving dietary pectin, with P2 presenting the lowest value. We hypothesize that supplemented diets could enhance the fish metabolism performance, enabling fish to use stored energy. Contrastingly, [23] demonstrated an increased in value of HSI in diets incorporated with 5 and 10 g kg^−1^ of pectin. The effects of prebiotics on the carcass’ chemical composition revealed no significant differences among treatments in terms of moisture, crude protein, crude lipid, and ash contents, whereas the 10 g kg^−1^ diet recorded the highest value of crude protein. The present results are consistent with the earlier results of [39,40] demonstrating increased protein contents in fish carcasses. In contrast to the present results, [41] exhibited a decrease in protein contents, as did [39] in the report of the effect of prebiotic diets on shrimp (*Penaeus semisulcatus*).

Digestive enzymes play a vital role in the metabolism of nutrients [42] and are essential factors in providing researchers with an indication of the most suitable dietary ingredients for their respective studies. We found, herein, that fish fed diets supplemented with pectin increased total protease and amylase activities, particularly in the P2 group, compared to the control diet. No significant differences were noticed among treatments in terms of lipase activity; however, the highest amount of lipase activity was observed in fish fed both the control (0 g) and 20 g kg^−1^ of pectin in their diets. Similar results were seen in *Sparus aurata*, fed a supplement of 4 g kg^−1^ of mannan-oligosaccharide compound, suggesting that prebiotics suitably modify gut microflora, resulting in superior physiological performance [43]. Furthermore, [44] exhibited that rainbow trout fed a diet supplemented with 2 g kg^−1^ of commercially available prebiotics (Immunogen), observed improvements in growth performance and digestive enzyme activity.

The present findings are in line with the findings in [43] that reported an increase in the digestive enzyme activity of rainbow trout fed 2 g kg^−1^ of Immunogen. Additionally, the research conducted by [40,45] determined that an enhancement of digestive enzyme activities, such amylase, lipase, and protease in common carp were achieved in diets containing short-chain fructo-oligo saccharides. Furthermore, [41] found that Japanese flounder (*Paralichthys olivaceus*) fed diets contain fructo-and mannan-oligosaccharide, enhanced digestive enzyme activity and lipid metabolism. The role of pectin in the enhancement of digestive enzyme activity may be attributed to (i) stimulation of the host gastrointestinal tract to secrete digestive enzymes, and (ii) increasein the level of external digestive enzymes secreted by the microbial flora of the gastrointestinal tract. 

More recently, [22] investigated the functions of pectin inclusion in the growth performance and immune response of common carp, in which pectin was proven to act as an immunostimulant, capable of enhancing the immune system and promoting growth. Additionally, [46] observed that the inclusion of pectin improved the intestinal morphology, leading to an improvement in the nutrient utilization efficiency and growth performance of fish. Furthermore, our results were consistent with those of [47], in which sea bream (*Sparus aurata*) larvae, fed pectin, improved both feed utilization and growth performance. The authors of [48] also demonstrated that Nile tilapia (*Oreochromis niloticus*) fed apple pectin demonstrated enhanced growth performance. The work in [49] reported that the use of dietary prebiotics, such as mannan-oligosaccharide, fructo-oligosaccharide, and galacto-oligosaccharide, led to an improvement in WG and SR in Atlantic salmon (*Salmo salar*). These findings may be explained by several different functions: pectin (i) modifies gut microflora community [50], (ii) improves digestive enzyme activities and absorption [7,51], (iii) modulates the stomach and intestinal structure [7]; (iv) encourages and enhances fish appetite [52]; (v) improves nutrient assimilation and removes pathogen microbes in fish gut [53]; (vi) generates a beneficial microflora community, that enhances the digestion and absorption of nutrients, and (vii) enhances the immune status and supports growth performance. The final product of prebiotic metabolism is a short chain of fatty acids that are assimilated by epithelial cells and can be used as an energy source to assist in the absorption of nutrients [25].

Ig, lysozyme, ACH50, described herein, are major elements that can improve immune responses and disease resistance in fish, through the break of carbohydrate molecules in the cell wall of microbes [54]. The highest values of Lysozyme were recorded in fish fed diets supplemented with 10 and 20 g kg^−1^ of APDP. Furthermore, diets supplemented with 5, 10, and 20 g kg^−1^ of APDP showed higher values of ACH50 compared to the control group. Additionally, the results showed that the level of total Ig was significantly increased in the pectin supplemented diets, compared to the control group. Similarly, [24,25] obtained an enhancement in serum lysosome and peroxidase in Nile tilapia (*Oreochromis nilticus*) fed pectin derived of orange peel. Furthermore, [24] observed that pectin had significant effects on the immune responses and disease outbreaks of Nile tilapia (*Oreochromis nilticus*). The authors [55] also determined that lemon-derived pectin enhanced immune system responses. The enhancement of the immune parameters, such as serum lysozyme, total Ig, and ACH50 in the present results, may be attributed to (i) immunostimulatory function of pectin [22]; (ii) improvement of different functions related to skin, gill, and gut lymphoid tissues [56]; (iii) improved secretion of natural antibodies [57], and (iv) antibacterial and antifungal activity [58].

The inclusion of pectin in the fish diets improved the TNF-α and IL-8 gene expressons compared to control fish. Our results showed that the mRNA levels in the groups fed with 5 and 10 g kg^−1^ of pectin supplementation were higher than that of the 20 g kg^−1^ group. No statistically significant differences (*p* ≥ 0.05) in IL-1β gene expression were detected between treatments as well as between treatments and the control. In terms of lysozyme gene expression, fish fed 10 and 20 g kg^−1^ pectin diets recorded the highest values of all groups. Our findings confirm the results of [59], which stated that more than 1200 gene expressions were altered significantly in diets supplemented with oligogalacturonide. Additionally, Velazquez et al. [60] also observed that the fish fed diets supplemented with mannan-oligosaccharides enhanced the gene expressions of anti- and pro-inflammatory cytokine gene expressions, including IL-1β, IL-6, IL-8, IL-10, IL-12, and IL-17 genes.

## 5. Conclusions

The present study highlights the importance of prebiotic inclusion in the diet of rainbow trout and its potential to improve growth performance and immune responses, as well as to stimulate the production of ACH50, lysozyme activity, and associated key immune gene expression. Thus, pectin could act as a natural feed additive in aquafeed to mitigate antibiotics and chemotherapeutics in aquaculture to reduce the antibiotic resistance phenomenon and to achieve a food free of antibiotics for human consumption.

## Figures and Tables

**Table 1:** Formulation and proximate composition of experimental diets incorporated with control: 0, P1: 5, P2: 10, and P3: 20 g of apple pomace derived pectin (APDP).

Diet Ingredients	Amount (g kg^−1^)			
	Control	P1	P2	P3
Fish meal ^1^	530	530	530	530
Corn flour ^2^	170	170	170	170
Soybean meal ^2^	107	107	107	107
Fish oil ^2^	105	105	105	105
Corn gluten 3	14	14	14	14
Mineral premix ^4^	10	10	10	10
Vitamin premix ^5^	10	10	10	10
Vitamin C ^6^	1	1	1	1
Binder ^7^	20	20	20	20
Choline chloride ^8^	7	7	7	7
Cellulose ^9^	20	15	10	0
Pectin	0	5	10	20
**Proximate Composition of Diets (%)**				
Crude protein	43.41	44	45.31	44.2
Crude lipid	11.1	10.88	10.65	11.05
NFE ^10^	16.2	17.65	18.37	18.88
Ash	11.1	11.25	11.15	11.35
Moisture	10.4	9.95	10.61	10.5
Gross energy (MJ.kg^−1^) ^11^	1437.522	1455.09	1481.337	1475.678

^1^ Anchou sprat meal, ^2^ provided by Khorak-e Dam Mazandaran, Sari, Iran, ^3^ provided by Glutenpars company; ^4^ mineral premix, amounts per kilogram of diet: mineral: Fe, 60 mg; Cu, 9 mg; Co, 0.7 mg; Se, 0.75 mg; Zn, 90 mg; Mn, 39 mg; I, 3 mg; ^5^ vitamin.kg^−1^ of diet: retinol acetate (A), 10,000 IU; cholecalciferol (D3), 6000 IU; DL-atocopheryl acetate (E), 600 mg; menadione sodium bisulfite (K3), 15 mg; D-Biotin (H2), 2.4 mg; thiamin mononitrate (B1), 45 mg; riboflavin (B2), 75 mg; calcium D-pantothenate (B3), 7200 mg; niacin amide (B5), 135 mg; pyridoxine hydrochloride (B6), 45 mg; folic acid (B9), 24 mg; cyanocobalamin (B12), 120 mg; antioxidant, 75 mg, ^6^ L-ascorbic acid (99%, Milwaukee, WI, USA); ^7^Amet binder^TM^, Mehr Taban-e-Yazd, Iran; ^8^ Sigma, Milwaukee, WI, USA); ^9^ a-cellulose (Sigma, Milwaukee, WI, USA); ^10^ Nitrogen free extract, ^11^ gross energy (MJ.kg^−1^ = (protein × 23.61 × 0.9) + (lipid × 39.82 × 0.85) + (NFE × 17.21 × 0.5).

**Table 2:** Sequence of primers used for gene expression.

Gene	Primers	Tissue	Access Number	Reference(s)
β-actin	Forward: AAAAAGCGCCAAAATAACAGAA	Reference Gene	AJ438158	Pérez-Sánchez et al. 2011
	Reverse: TCCGACGGTAAACATCCTTC			
IL-1β	Forward: ACATTGCCAACCTCATCATCG	Kidney	AJ223954	Pérez-Sánchez et al. 2011
	Reverse: TTGAGCAGGTCCTTGTCCTTG			
IL-8	Forward: AGAATGTCAGCCAGCCTTGT	Kidney	AJ279069	Pérez-Sánchez et al. 2011
	Reverse: TCTCAGACTCATCCCCTCAGT			
TNF-α	Forward: GGGGACAAACTGTGGACTGA	Kidney	AJ277604	Pérez-Sánchez et al. 2011
	Reverse: GAAGTTCTTGCCCTGCTCTG			
Lyz	Forward: ACAGCCGCTACTGGTGTGACG	Kidney	X59491.1	Taheri Mirghaed et al., 2019
	Reverse: GCTGCTGCCGCACATAGAC			

**Table 3:** Growth performance of rainbow trout fed four experimental diets with different inclusion levels control: 0, P1: 5, P2: 10, and P3: 20 g of apple pomace derived pectin (APDP) for 30 days. Values are presented as the mean ± SE.

Parameters	Control	P1	P2	P3
IW (g)	3.58 ± 0.01	3.57 ± 0.02	3.55 ± 0.01	3.53 ± 0.01
FW (g)	14.16 ± 0.02 ^c^	18.11 ± 0.05 ^a^	18.22 ± 0.04 ^a^	17.87 ± 0.07 ^b^
WG (g)	10.58 ± 0.03 ^c^	14.54 ± 0.05 ^ab^	14.67 ± 0.02 ^a^	14.33 ± 0.07 ^b^
SGR (%/d^−1^)	3.27 ± 0.01 ^b^	3.86 ± 0.01 ^a^	3.89 ± 0.01 ^a^	3.85 ± 0.01 ^a^
FCR	1.49 ± 0.005 ^a^	1.08 ± 0.002 ^c^	1.07 ± 0.001 ^d^	1.10 ± 0.005 ^b^
HSI (%)	2.21 ± 0.06 ^a^	1.28 ± 0.04 ^c^	0.96 ± 0.05 ^d^	1.58 ± 0.05 ^b^
VSI (%)	9.98 ± 0.09 ^a^	6.73 ± 0.04 ^c^	5.99 ± 0.03 ^d^	7.16 ± 0.11 ^b^

IW: initial weight; FW: final weight; WG: weight gain; SGR: specific growth rate; FCR: feed conversion ratio; SR: survival rate, HSI: hepato somatic index, VSI: viscera somatic index. Different letters (a–d) in the same row indicate significant differences (*p* ≤ 0.05).

**Table 4:** Body compositions of rainbow trout fed four experimental diets with different inclusion levels control: 0, P1: 5, P2: 10, and P3: 20 g of apple pomace derived pectin (APDP) for 30 days. Values are presented as the mean ± SE.

Parameters (%)	Control	P1	P2	P3
Moisture	69.62 ± 1.20	70.49 ± 1.18	71.04 ± 1.40	70.63 ± 0.74
Crude protein	15.15 ± 0.93	16.41 ± 1.17	16.97 ± 1.32	16.73 ± 0.80
Crude lipid	4.33 ± 0.14	4.19 ± 0.04	4.09 ± 0.03	4.37 ± 0.10
Ash	3.19 ± 0.12	3.38 ± 0.17	3.24 ± 0.15	3.41 ± 0.15

**Table 5:** Digestive enzymes activity of rainbow trout fed four experimental diets with different inclusion levels control: 0, P1: 5, P2: 10, and P3: 20 g of apple pomace derived pectin (APDP) for 30 days. Values are presented as the mean ± SE.

Parameters	Control	P1	P2	P3
Protease (U/mg pro)	1.21 ± 0.03 ^b^	1.34 ± 0.02 ^a^	1.44 ± 0.03 ^a^	1.38 ± 0.02 ^a^
Amylase (U/mg pro)	0.58 ± 0.03 ^b^	0.72 ± 0.02 ^a^	0.82 ± 0.03 ^a^	0.74 ± 0.02 ^a^
Lipase (U/mg pro)	0.31 ± 0.03	0.27 ± 0.02	0.26 ± 0.02	0.33 ± 0.02

Different letters (a–b) in the same row indicate significant differences (*p* ≤ 0.05).

## Data Availability

Data are available on request due to restrictions, e.g., privacy or ethical. The data presented in this study are available on request from the corresponding author. The data are not publicly available due to the law of the Ministry of Science Research and Technology.
